# Supplementary material for: Fibrous network nature of plant cell walls enables tunable mechanics for development
Source: Nat Commun. 2025 Aug 14;16:7565. doi: 10.1038/s41467-025-62844-1 (PMC12354786; doi:10.1038/s41467-025-62844-1)
Supplement: Supplementary file 1 — Supplementary Information [file 41467_2025_62844_MOESM1_ESM.pdf]

Fibrous Network Nature of Plant Cell Walls Enables Tunable Mechanics for Development

Si Chen, Isabella Burda, Purvil Jani, Bex Pendrak,
Meredith N. Silberstein, and Adrienne Roeder

Supplementary Notes

1 Two-dimensional affine isotropic network model

The two dimensional affine isotropic network model[1] is based on averaging the response of a single fiber to an applied deformation over all orientations of randomly distributed fibers in a plane. We assume that all fibers only support force along their current orientation axis (i.e. no shear) and are only able to stretch or compress in that same axis. A fiber with initial orientation $\hat{n} = (\cos\theta, \sin\theta)$, which is subjected to deformation gradient tensor $\mathbf{\Lambda}$, will deform axially and rotate. The force within the fiber is given by $k(|\mathbf{\Lambda}\hat{\mathbf{n}}| - 1)$, where k is the stiffness of the fiber and set to 1 for simplicity. The new orientation of the fiber would be $(\mathbf{\Lambda}\hat{\mathbf{n}})/|\mathbf{\Lambda}\hat{\mathbf{n}}|$. Taking the average over all orientations, we have the membrane stress tensor of the network:

$$\begin{aligned}\sigma_{ij} &= \frac{\rho}{\det\mathbf{\Lambda}}\left\langle k(|\mathbf{\Lambda}\hat{\mathbf{n}}| - 1)\frac{\Lambda_{il}n_l\Lambda_{jm}n_m}{|\mathbf{\Lambda}\hat{\mathbf{n}}|}\right\rangle \\ &= \frac{\rho}{2\pi\det\mathbf{\Lambda}}\int_\theta k(|\mathbf{\Lambda}\hat{\mathbf{n}}| - 1)\frac{\Lambda_{il}n_l\Lambda_{jm}n_m}{|\mathbf{\Lambda}\hat{\mathbf{n}}|}d\theta\end{aligned} \tag{1}$$

where ρ is fiber length density (length per unit of area), set to $\frac{1}{2\pi}$ for simplicity.

In the uniaxial tension case, consider applying $\mathbf{\Lambda} = \begin{pmatrix}\lambda_1 & 0\\ 0 & \lambda_2\end{pmatrix}$, where λ_1 is prescribed value and λ_2 needs to be solved such that $\sigma_{22} = 0$. Here

$$|\mathbf{\Lambda}\hat{\mathbf{n}}| = \sqrt{\lambda_1^2\cos^2\theta + \lambda_2^2\sin^2\theta} \tag{2}$$

$$\det\mathbf{\Lambda} = \lambda_1\lambda_2 \tag{3}$$

The stress tensor for the fiber network is then given by:

$$\sigma_{11} = \frac{1}{\det\mathbf{\Lambda}}\int_\theta \frac{(|\mathbf{\Lambda}\hat{\mathbf{n}}| - 1)\lambda_1^2\cos^2\theta}{|\mathbf{\Lambda}\hat{\mathbf{n}}|}d\theta \tag{4}$$

$$\sigma_{22} = \frac{1}{\det\mathbf{\Lambda}}\int_\theta \frac{(|\mathbf{\Lambda}\hat{\mathbf{n}}| - 1)\lambda_2^2\sin^2\theta}{|\mathbf{\Lambda}\hat{\mathbf{n}}|}d\theta \tag{5}$$

$$\sigma_{12} = \frac{1}{\det\mathbf{\Lambda}}\int_\theta \frac{(|\mathbf{\Lambda}\hat{\mathbf{n}}| - 1)\lambda_1\lambda_2\cos\theta\sin\theta}{|\mathbf{\Lambda}\hat{\mathbf{n}}|}d\theta \tag{6}$$

To solve $\sigma_{22} = 0$ numerically, we assume two initial values for λ_2: $\lambda_{2_{t0}}$ and $\lambda_{2_{t1}}$, and calculate corresponding $\sigma_{22_{t0}}$ and $\sigma_{22_{t1}}$. We use the Secant method to find the next guess value:

$$\lambda_{2_{t2}} = \lambda_{2_{t1}} - \sigma_{22_{t1}}\frac{\lambda_{2_{t1}} - \lambda_{2_{t0}}}{\sigma_{22_{t1}} - \sigma_{22_{t0}}} \tag{7}$$

We repeat this process until $\sigma_{22} < 10^{-10}$, so that we find the λ_2. Then, the stress tensor can be calculated based on λ_1 and λ_2 using Eqns. 4, 5 and 6. The force per width (nominal stress) can be calculated by $S_{11} = \sigma_{11}\lambda_2$.

2 Five-beam model

In the five-beam model[8], there are five beams, and they are linked by connectors that transmit force, but not moments, between beams. When the structure deforms, the four tilted beams AC, AD, BC, and BD will rotate and stretch, the beam AB will deform only through bending, and the connectors will undergo shear deformation and slide along the beam if the load exceeds a certain threshold (Supplementary Fig. 11a). We first look at the force-deformation relationship of each component respectively.

Stretching beam with stretching stiffness k_s

These beams are elongated elastically under stretching force F_s. The elongation length is given by $\Delta L_s = F_s/k_s$ (Supplementary Fig. 11b).

Bending Beam with bending stiffness k_b

This beam is bent elastically under compression force F_b. The end of beam moves distance $\Delta L_b = F_b/k_b$ (Supplementary Fig. 11c).

Connector with shear stiffness k_c and sliding resistance D

The connector is under shear when two connected beams are pulled apart from each other. Two kinds of displacement need to be considered in this case. First, there is elastic deformation of the connector resulting in displacement $\Delta u_c = F_c/k_c$. Then, fibers are allowed to slide relative to each other along the connector, resulting in displacement $\Delta u_{slide} = (F_c - F_0)/D$, only when the force F_c exceeds the threshold F_0. During unloading the elastic deformation of the connector can fully recover, while the sliding distance cannot recover (*i.e.,* Δu is set according to the maximum F_c value experience during the loading history). Therefore, the total displacement is (Supplementary Fig. 11d): If reloading, the sliding distance remains unchanged until the load exceeds the maximum load F_m in history. The maximum load F_m serves as a new yield force threshold. After exceeding the maximum load, the sliding distance still follows the equation: $\Delta u_{slide} = (F_c - F_0)/D$. Therefore, the total displacement

$$\Delta u_c + \Delta u_{slide} = h(F_c) = \begin{cases} \frac{F_c}{k_c} + \text{const} & \text{if during unloading or if loading with } F_c < F_m \\ F_c(\frac{1}{k_c} + \frac{1}{D}) - \frac{F_0}{D} & \text{if loading with } F_c > F_m \end{cases} \tag{8}$$

where h is the piecewise function.

Consider a five-beam structure, the initial length of each of the four bending beams is the same, denoted as L_{b0}, and the initial tilt angle of the stretching beam is θ_0, so the initial length of each the stretching beams is $L_{s0} = \frac{L_{b0}}{2\sin\theta_0}$. A force F in x direction is prescribed on the node C and D of the five-beam structure. According to force balance relations, the same force acts on each of the stretching beams:

$$F_s = \frac{F}{2\cos\theta} \tag{9}$$

and the force acting on the bending beam:

$$F_b = F\tan\theta \tag{10}$$

and the force acting on the connectors:

$$F_c = F_s = \frac{F}{2\cos\theta} \tag{11}$$

where θ is the current tilt angle of the stretching beams.

According to the geometric relation, we have the distance between nodes A and C:

$$L_{AC} = L_{s0} + \Delta L_s + \Delta u_c + \Delta u_{slide} \tag{12}$$

the distance between nodes A and B:

$$L_{AB} = L_{b0} - \Delta L_b - \Delta u_c - \Delta u_{slide} \tag{13}$$

the distance between node C and D:

$$L_{CD} = 2L_{AC}\cos\theta \tag{14}$$

L_{AB} and L_{AC} should satisfy

$$2L_{AC}\sin\theta = L_{AB} \tag{15}$$

According to force-displacement relations discussed above, we have

$$\Delta L_s = F_s/k_s \tag{16}$$

$$\Delta L_b = F_b/k_b \tag{17}$$

$$\Delta u_c + \Delta u_{slide} = h(F_c) \tag{18}$$

So far we have 10 unknowns: θ, F_s, F_b, F_c, ΔL_s, ΔL_b, $\Delta u_c + \Delta u_{slide}$, L_{AC}, L_{AB}, L_{CD}, which can be solved from the above ten Eqns. 9-18.

To increment the applied force F by dF in each step, we use the incremental form of these equations:

$$dF = 2\cos\theta dF_s - 2F_s\sin\theta d\theta \tag{19}$$

$$dF_b = dF\tan\theta + \frac{Fd\theta}{\cos^2\theta} \tag{20}$$

$$dF_c = dF_s \tag{21}$$

$$dL_{AC} = d\Delta L_s + d\Delta u_c + d\Delta u_{slide} \tag{22}$$

$$dL_{AB} = -d\Delta L_b - d\Delta u_c - d\Delta u_{slide} \tag{23}$$

$$dL_{CD} = 2dL_{AC}\cos\theta - 2L_{AC}\sin\theta d\theta \tag{24}$$

$$2dL_{AC}\sin\theta + 2L_{AC}\cos\theta d\theta = dL_{AB} \tag{25}$$

$$d\Delta L_s = \frac{dF_s}{k_s} \tag{26}$$

$$d\Delta L_b = \frac{dF_b}{k_b} \tag{27}$$

$$d\Delta u_c + d\Delta u_{slide} = \frac{dh(F_c)}{dF_c}dF_c \tag{28}$$

Combining Eqns. 19 - 28, we obtain:

$$-\frac{dF_b}{k_b} - \frac{dh(F_b)}{dF_b}dF_b = 2\left(\frac{dF_s}{k_s} + \frac{dh(F_s)}{dF_s}dF_s\right)\sin\theta + 2L_{AB}\cos\theta d\theta \tag{29}$$

Note here θ and L_{AC} are measured in current configuration after dF is loaded in current step, and can be estimated as θ_p and L_{AC_p} in the previous step if dF is very small. Then dF_b, dF_s and $d\theta$ can be solved from Eqns. 19, 21 and 29.

Finally, the incremental stretches of the five-beam structure can be calculated as:

$$d\lambda_x = \frac{dL_{CD}}{L_{CD_0}} = \frac{dL_{AC}\cos\theta - L_{AC}\sin\theta d\theta}{L_{s0}\cos\theta_0} \tag{30}$$

$$d\lambda_y = \frac{dL_{AB}}{L_{b0}} \tag{31}$$

with total stretches of:

$$\lambda_x = \frac{L_{AC}\cos\theta}{L_{s0}\cos\theta_0} - 1 \tag{32}$$

$$\lambda_y = \frac{L_{AB}}{L_{b0}} - 1 \tag{33}$$

The plant cell walls exhibit a characteristic diamond-shaped arrangement of cellulose microfibrils and the distance between adjacent cellulose microfibrils is between 20 to 150 nm[9]. The thickness of microfibrils is around 3.6 ± 1.9 nm[3]. So we chose the stretching stiffness and bending stiffness ratio $\frac{k_s}{k_b} = \frac{Al^2}{3I} \sim \frac{l^2}{d^2} \sim 100$, where A and I represent the area and the second moment of the area. For a qualitative comparison, we chose the following parameters. We set $k_b = 1$ so that $k_s = 100$. The length of the beam simply scales the overall force-displacement response, so we made the arbitrary choice of $l_{b0} = 10$. The initial angle θ_0 will influence the incremental Poisson's ratio. We chose $\theta_0 = \frac{1.3\pi}{4}$ so that the initial incremental Poisson's ratio of the model was within the same range as the experiments. We chose $k_c = 36$ and $D = 24$ so that the stiffening ratio and recovery ratio were comparable to the experiments on the 25d samples. The connector resistance was calculated as $C = \frac{1}{\frac{1}{D}+\frac{1}{k_c}}$. Different connector resistance values C_1, C_2 and C_3 were corresponding to three sets of chosen value (1) $k_c = 15$ and $D = 10$; (2)$k_c = 30$ and $D = 20$; (3) $k_c = 36$ and $D = 24$ respectively. Alternative initial angle values of $\theta_1 = \frac{1.2\pi}{4}$ and $\theta_2 = \frac{1.4\pi}{4}$ were chosen to demonstrate possible anisotropic effects.

3 Turgor pressure and cell deformation relation under linear and stiffening behavior

Considering a spherical cell with initial radius r_0, assume the cell wall is isotropic, so that the cell will expand to new radius r under turgor pressure P (Supplementary Fig. 12a). The stretch ratio of the cell wall $\lambda = \frac{r}{r_0}$. According to Laplace's law, the force per width within cell wall is

$$T = \frac{Pr}{2} \tag{34}$$

Case I

In linear mechanical behavior case, the force per width within the cell wall would always be proportional to the stretch, in other word, the stiffness E is constant. Therefore, we have force per width and stretch relation as:

$$T = E(\lambda - 1) \tag{35}$$

Combining Eqns. 34 and 35, we have turgor pressure and stretch relation as:

$$P = E\frac{\lambda - 1}{\lambda}\frac{2}{r_0} \tag{36}$$

Case II

In the nonlinear stiffening case, we consider the stiffness $E = E(\lambda)$ as a function of the stretch. Therefore, we have force per width and stretch relation in incremental form as:

$$\delta T = E(\lambda)\delta\lambda \tag{37}$$

Combining Eqns. 34 and 37, we have turgor pressure and stretch relation as incremental form as:

$$\delta P = \frac{2\delta T}{\lambda r_0} - \frac{2T\delta\lambda}{\lambda^2 r_0} \tag{38}$$

We consider the initial cell size is 10 µm. For **case I**, the stiffness $E = 14.43$ (Supplementary Fig. 12b). For **case II**, we use a sigmoid function from fitting the stiffness versus stretch curve in Supplementary Fig. 1e,

which is $E(\lambda) = E_0 + \frac{E_1 - E_0}{1+e^{-a(x-b)}} = 14.43 + \frac{51.71}{1+e^{-58.29(x-1.119)}}$ (Supplementary Fig. 12b). The turgor pressure and stretch curves for both cases are plotted in Supplementary Fig. 12c.

4 Mechanical behavior under general loading conditions

In this section, we will derive the deformation of the cell wall under general loading conditions using results from tensile tests. We will consider both linear and nonlinear elasticity formulations.

4.1 Linear isotropic elastic formulation

Linear elasticity formulation can be used under the infinitesimal deformation assumption[10], where the deformation changes linearly with applied force. Since cell wall is very thin and its mechanical properties are similar within the plane from our tensile test results, we consider two-dimensional isotropic elastic theory using plane stress state[10], the deformation under loading can be characterized from Hooke's law:

$$\begin{aligned} \epsilon_x &= \frac{1}{E}(s_x - \nu s_y) \\ \epsilon_y &= \frac{1}{E}(s_y - \nu s_x) \end{aligned} \tag{39}$$

where ϵ_x and ϵ_y are strains in x and y directions respectively, defined as the stretch in each direction minus 1. s_x and s_y are forces per width in x and y directions respectively. E and ν are Young's modulus and Poisson's ratio respectively.

From tensile tests described in the main text, we have $s_y = 0$. Therefore, $E = s_x/\epsilon_x$ and $\nu = -\frac{\epsilon_y}{\epsilon_x}$. Once E and ν are determined, the deformation of the cell wall (ϵ_x and ϵ_y) can be calculated under any loading conditions (varying s_x and s_y). We will consider two types of loading conditions that are closely related to what cell walls experience in a live cell and demonstrate how to calculate the resulting deformation.

Case I: equal-biaxial loading The cell wall of a spherical cell (radius r) is subjected to equal-biaxial loading due to the turgor pressure P. According to Laplace's law, forces per width within the cell wall are: $s_x = s_y = \frac{Pr}{2}$. Therefore, the deformation can be calculated according to Eqn. 39: $\epsilon_x = \epsilon_y = \frac{1-\nu}{E}\frac{Pr}{2}$. From this result, we can see that a larger Poisson's ratio makes cell wall more resistant to deform under equal-biaxial loading.

Case II: turgid cylindrical cell under stretch Considering a cylindrical cell with turgor pressure P, length l_0 and radius r_0. The cell is stretched by an axial force δF (Supplementary Fig. 13a), resulting in length change δl and radius change δr. Here we assume turgor pressure is kept as constant. The force per width in axial direction $\delta T_a = \frac{P\delta r}{2} + \frac{\delta F}{2\pi r_0}$, and in transverse(circumferential) direction $\delta T_h = P\delta r$. The strain in axial direction $\epsilon_a = \frac{\delta l}{l_0}$, and in transverse direction $\epsilon_h = \frac{\delta r}{r_0}$. Combining Eqn. 39, we can calculate the stiffness of this cell as: $E_{cell} = \frac{\delta F/\pi r_o^2}{\epsilon_a} = \frac{2E(2E - Pr_0(2-\nu))}{r_0(2E - Pr_0(1-\nu^2))}$. If we take Young's modulus $E = 20$ N/m, we have turgor pressure $P = 0.5$ MPa, and the radius $r = 10$ µm, and plot E_{cell} versus ν (Supplementary Fig. 13b). From this plot, a larger Poisson's ratio leads a larger stiffness E_{cell}, indicating that the cell becomes more resistant to forces from its environment or neighboring cells.

4.2 nonlinear elastic formulation

The cell wall has a polylamellate structure[13], which results in different mechanical properties along the thickness direction compared to within the plane of the cell wall. From our tensile test results, mechanical properties are similar within the cell wall plane. Therefore, we consider a nonlinear transversely isotropic (plane isotropy) formulation based on hyperelastic constitutive theories[4,11]. Given that the cell wall matrix, composed of pectin and hemicellulose, is hydrogel-like, we assume the cell wall is incompressible (volume-conserving). We assume the strain energy(Helmholtz free energy) form[11]: $W = w(I_1, \lambda_3) - p(J-1)$, where $I_1 = \lambda_1^2 + \lambda_2^2 + \lambda_3^2$, $J = \lambda_1\lambda_2\lambda_3 = 1$, p introduced serves as an indeterminate Lagrange multiplier, λ_1 and λ_2 are the stretches in two perpendicular directions within the cell wall plane, and λ_3 is the stretch along the thickness direction. The strain energy function fully characterizes the mechanical behavior, allowing us to

determine the relationship between force and deformation. Therefore, we have

$$\begin{aligned} s_1 &= 2\lambda_1\beta - p\lambda_2\lambda_3 \\ s_2 &= 2\lambda_2\beta - p\lambda_1\lambda_3 \\ s_3 &= 2\lambda_3\beta + \alpha - p\lambda_2\lambda_3 \end{aligned} \tag{40}$$

where $\beta = \frac{\partial w}{\partial I_1}$ and $\alpha = \frac{\partial w}{\lambda_3}$. From tensile tests described in the main text, we have $s_2 = 0$, $s_3 = 0$. s_1, λ_1, and λ_2 are measured, and $\lambda_3 = \frac{1}{\lambda_1\lambda_2}$. So that we have $\beta = \frac{s_1\lambda_1}{2(\lambda_1^2-\lambda_2^2)}$ and $\alpha = \frac{2\beta(\lambda_2^2-\lambda_3^2)}{\lambda_3}$. We assume that the strain energy does not include cross-terms involving I_1 and λ_3, so β only depends on I_1 and α only depends on λ_3. Therefore, these functions can be determined respectively. Then we will explore relation between force and deformation under equal-biaxial loading based on Eqn. 40.
equal-biaxial loading We have $\lambda_1 = \lambda_2 = \lambda_b$ and $\lambda_3 = \lambda_t$. Forces per width in plane $s_1 = s_2 = s = 2\beta_1\lambda_b - p\lambda_b\lambda_t$, where $p = \frac{2\beta\lambda_t+\alpha}{\lambda_b^2}$.$\beta$ and α can be determined based on $I_1 = 2\lambda_b^2\lambda_t$ and $\lambda_3 = \lambda_t$ separately. This give s versus λ_b curves for equal-biaxial loading as shown in Supplementary Fig. 14.

Methods

1 Tensile tests

1.1 Other plant material

The *qua2-1* (AT1G78240) mutant in the Col-0 background is used[7,12]. The first two true leaves were dissected for tensile tests on post-germination day 26.

1.2 Custom micromechanical tensile stage

The custom tensile stage is shown as a 3D CAD representation in Supplementary Fig. 15. This stage includes the following components: (1) Steel bottom plate; (2) Newport MS-125-XYZ miniature linear stage; (3) Aluminum connection plate1; (4) Newport AG-LS25 piezo motor driven linear stage; (5) Aluminum connection plate 2; (6) Aluminum rod fixture; (7) L-shape fixture; (8) Futek LSB200 load cell; (9) Aluminum L-shape connection.

1.3 Leaf epidermal peel preparation

A mixture of 5g gelatin (Fisher, G8500) and 10g water was heated until the gelatin dissolved completely and then maintained at 80°C. The mixture was applied to a piece of cloth, and a dissected leaf was placed quickly onto the gelatin-applied area (Supplementary Fig. 8a). The cloth was folded and gently pressed to ensure the gelatin mixture made full contact with both surfaces of the leaf. After a half-hour curing process at room temperature, the folded cloth was unfolded by quickly pulling apart the two ends, separating the abaxial epidermal peel from the leaf (Supplementary Fig. 8b). A soft wet brush was used to gently sweep the peeled surface to remove any mesophyll cells attached to the abaxial epidermal peel. The cloth was then immersed in water for 20 seconds to reduce adhesion between the leaf and gelatin. Finally, the leaf epidermal peel was isolated from the cloth using tweezers. For forzen and thawed treatment samples, the leaf epidermal peels were transfered to -80°C overnight and then thawed in the room-temperature water prior to testing.

1.4 Fluorescent bead dilution

We diluted the fluorescent bead suspension with Milli-Q Water by factors of 10, 100, and 500. The different concentrations did not have a noticeable influence on the mechanical behavior of the epidermal sample. A dilution factor of 100 provided the best pattern for DIC analysis.

1.5 Staining epidermis with calcofluor white

Place peeled epidermis into Eppendorf tube with 2 mg/mL calcofluor solution, then put the tube under vacuum for 10 minutes. Rinse the tissue with water before imaging.

1.6 Choice of force per width as a measurement unit

In this study, we report force per unit width (N/m) rather than stress (Pa) for the following reasons. Plant primary cell wall thickness stays relatively constant in most cases as the cell grows, since wall synthesis and wall expansion are well-coordinated[2,6]. This suggests that samples in our tests have similar wall thickness, so force per width reflects intrinsic mechanical stiffness. In addition, the use of force per width avoids the need to measure wall thickness directly, which may be impractical to measure consistently since the cell wall is extremely thin and its thickness varies within a single cell. Furthermore, force per width (N/m) allows a direct connection between experimental measurements and the biophysical model that relates turgor pressure to cell wall deformation[5] without requiring explicit thickness measurements. From tensile tests, we obtained how much the cell wall deforms under a given level of force per width, captured by the force per width-stretch relationship: force per width = stiffness * (stretch -1). This relation can be used to relate turgor pressure to cell wall deformation in a turgid cell. Within a cell, the balance of forces gives turgor pressure = stiffness * (stretch -1) * perimeter of cross section/ cross-sectional area of the cell, without acquiring explicit thickness. In general, we note the use of stiffness in N/m is appropriate to understand the intrinsic cell wall material properties only when there is no substantial variation in wall thickness across samples.

2 Scanning Electron Microscopy (SEM) imaging

Leaf epidermal peels were obtained as described in the tensile test methods and mounted to coverglass with the inner surface facing up. The peels were fixed in FAA (50% ethanol, 3.7% formaldehyde, 5% acetic acid) for 4 hours at room temperature. Following fixation, the peels were subjected to a series of increasing ethanol concentrations (50%, 50%, 60%, 70%, 80%, 90%, 95%, 100%, 100%, 100%) for 30 minutes each at room temperature and kept in 100% ethanol overnight at 4°C. On the second day, the peels were critical point dried and sputter-coated with gold palladium for 2 minutes to a thickness of approximately 40 nm. SEM imaging was performed using a Zeiss Gemini 500 Scanning Electron Microscope with EHT = 1.00 kV.

3 Finite element simulations

3.1 Extracting the cellular structure from the confocal images

First, the maximum intensity Z projection image was obtained from the Zeiss z-stack image. This projection image was then segmented using Watershed segmentation from the MorphoLibJ plugin in ImageJ software. The outline of the cells was traced (Centerline tracing) from the segmented image, and any gaps were fixed using the Snap Object Points plugin and manual adjustments. The resulting cellular structure was exported as a .dxf file in Inkscape, which was then imported into ABAQUS Standard(2018). In ABAQUS, the cellular structure was constructed by extruding the cell outline to form the anticlinal walls, and the flat top and bottom periclinal walls were connected to the anticlinal walls using tie constraints. No material was assigned to the cell lumens to reflect the absence of turgor pressure.

3.2 Finite element type

The constructed cellular structure was meshed with 4-node reduced integration shell elements (S4R with hourglass control and finite membrane strains) with thickness of cell walls corresponding to thickness of the shell. The seed distance was set to 4 µm for the top/bottom walls and 2 µm for the side walls, based on convergence studies.

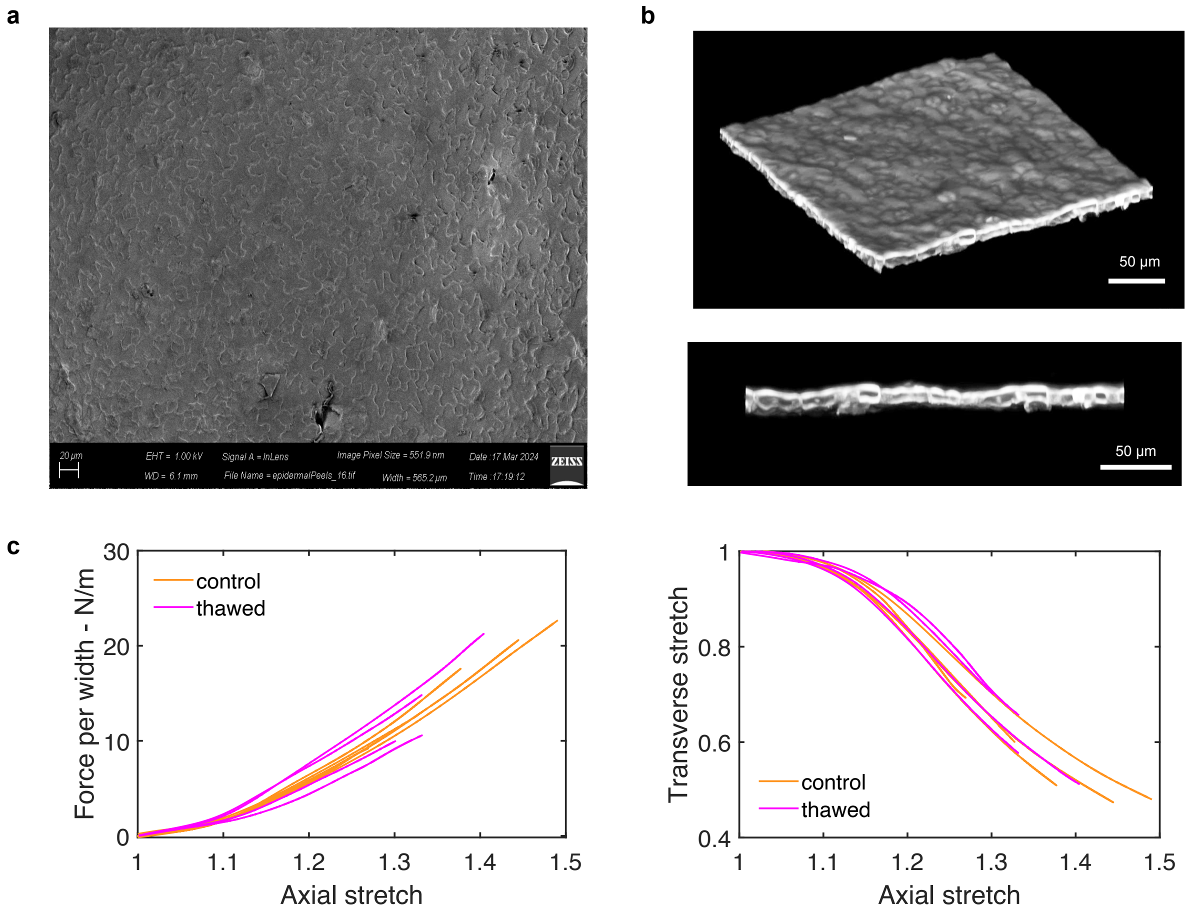

Supplementary Figure 1. **Epidermal peel is a layer of deflated intact epidermal cells.** **a** SEM image of leaf epidermal peel with the inner surface facing upwards. Note the residual debris from mesophyll cells (Supplementary Methods 2). **b** 3D confocal images of a sample stained by calcofluor white in isometric view and side view (Supplementary Methods 1.5). **c** Force per width – axial stretch and transverse – axial stretch curve for control as well as frozen and thawed samples at 18-day stage. Note that four curves from control tests are reused from Fig. 3B 18d data.

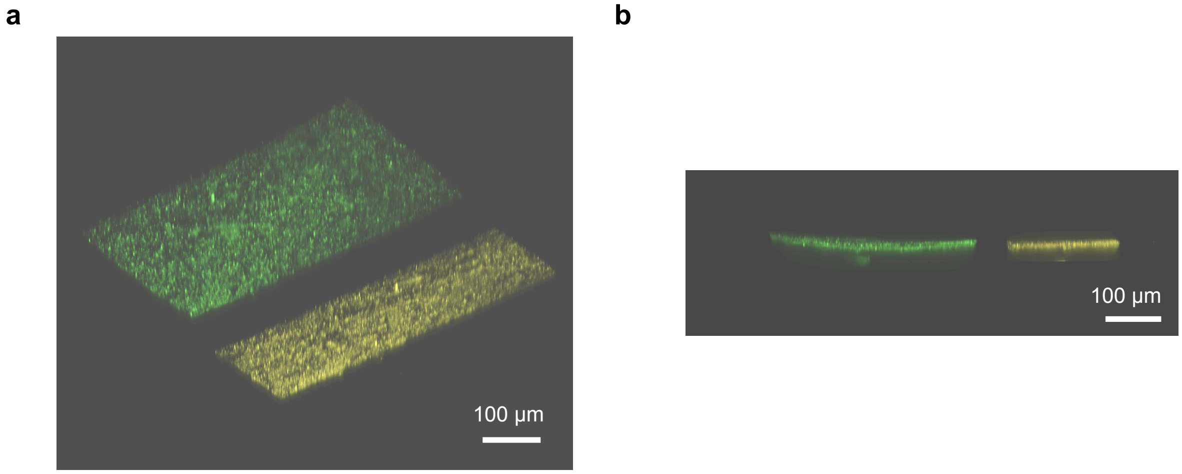

Supplementary Figure 2. **3D confocal images of a sample coated with fluorescent beads in undeformed (green) and deformed (yellow) states, showing no obvious wrinkling.** **a** Isometric view; **b** side view along the sample width.

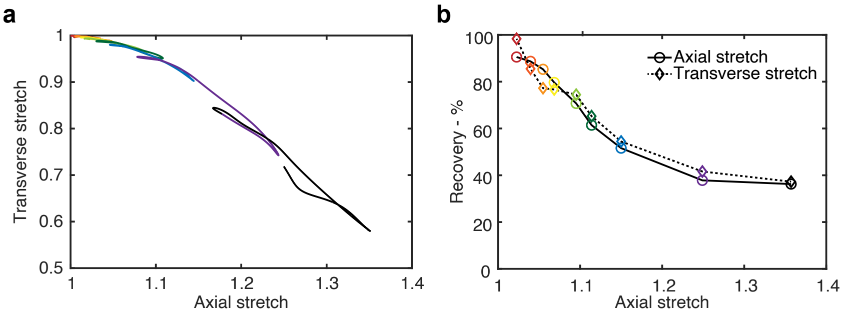

Supplementary Figure 3. **Transverse stretch change from the same incremental cyclic tensile test shown in Fig. 1h.** **a** Transverse stretch versus axial stretch curve. **b** Recovery of transverse stretch and axial stretch, each plotted against axial stretch to compare their respective behaviors.

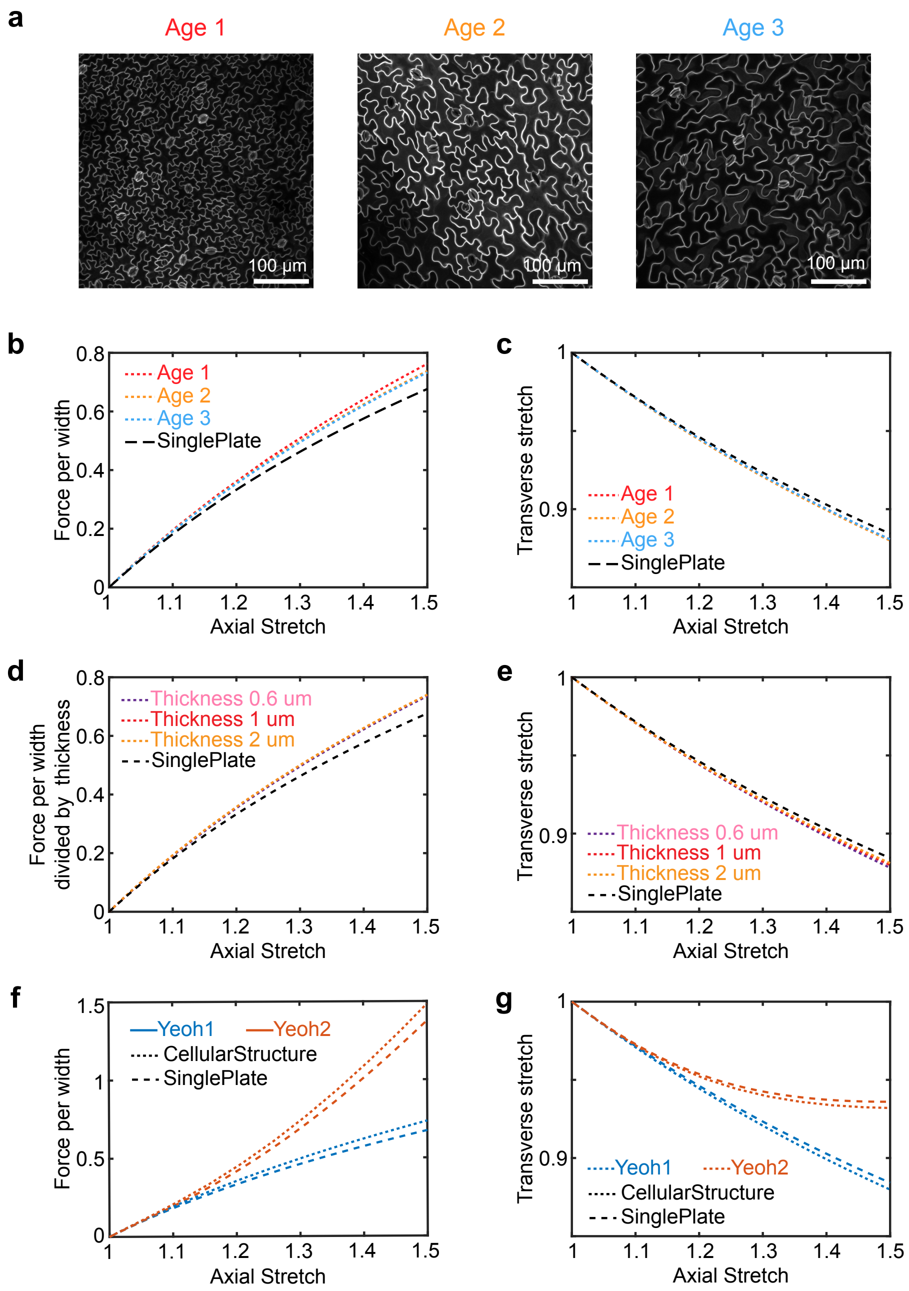

Supplementary Figure 4. **Cellular structure has little impact on overall mechanical behavior in cases of varying the cell size of the cellular structure, the thicknesses of the cell wall, and the material models of the cell wall.** **a** Confocal images of abaxial epidermal cells from leaves at various developmental stages showing different cell sizes: small (12d), medium (18d), and large (25d) cell sizes; **b** Force per width versus axial stretch curves and **c** Transverse stretch versus axial stretch curves of FEM simulation of three cellular structures constructed from three configuration and a single plate; **d** Force per width versus axial stretch curves and **e** Transverse stretch versus axial stretch curves of FEM simulation of cellular structures with three different thicknesses and a single plate; **f** Force per width versus axial stretch curves and **g** Transverse stretch versus axial stretch curves of FEM simulation of a cellular structure and a single plate using the Yeoh hyperelastic model with two different material parameters.

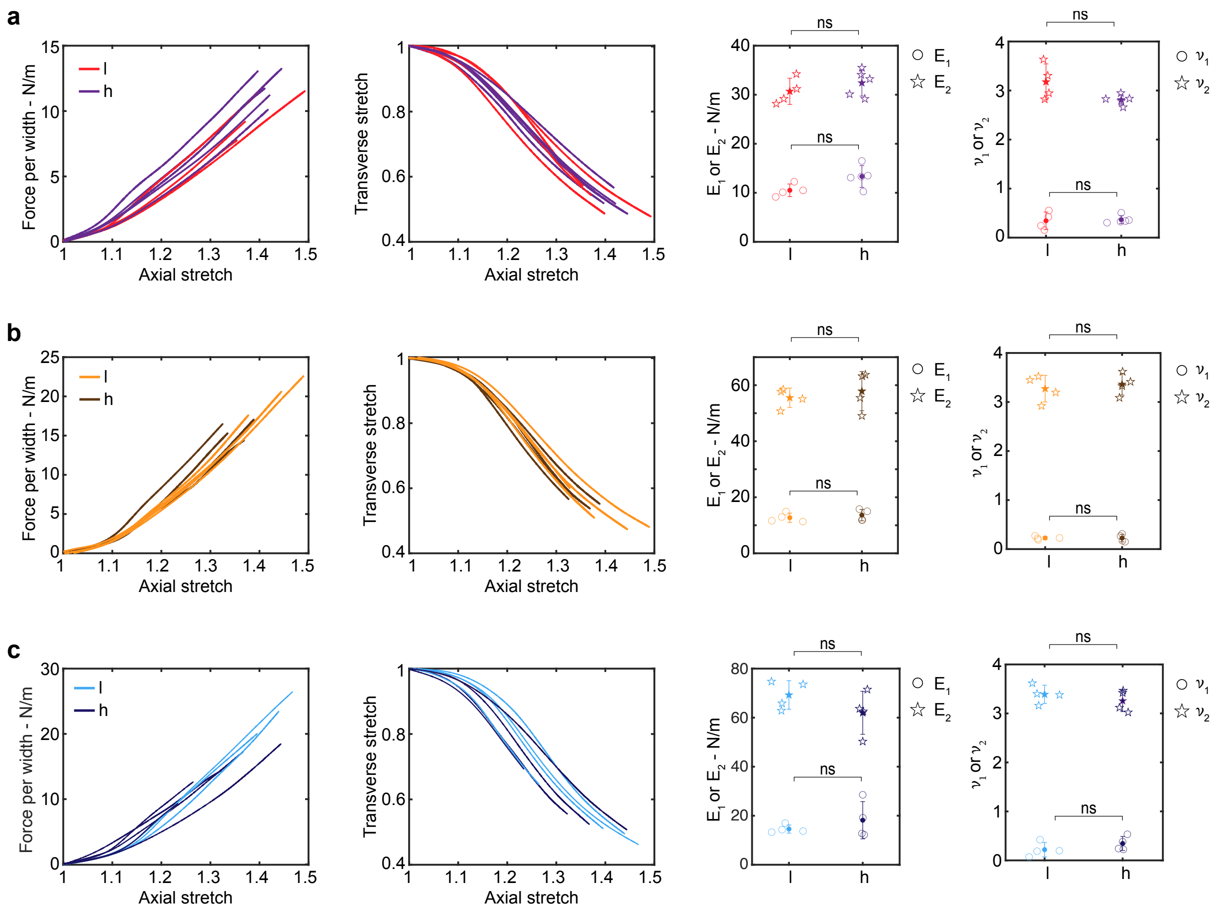

Supplementary Figure 5. **Comparison of monotonic tensile test results for wild type in proximal-distal (l) and medial-lateral (h) directions at three developmental stages: a** 12 days, **b** 18 days, and **c** 25 days. This includes force per width versus axial stretch curves, transverse stretch versus axial stretch curves, initial stiffness (E_1), final stiffness (E_2), initial Poisson's ratio (ν_1), and peak Poisson's ratio (ν_2), compared between directions for each stage respectively.

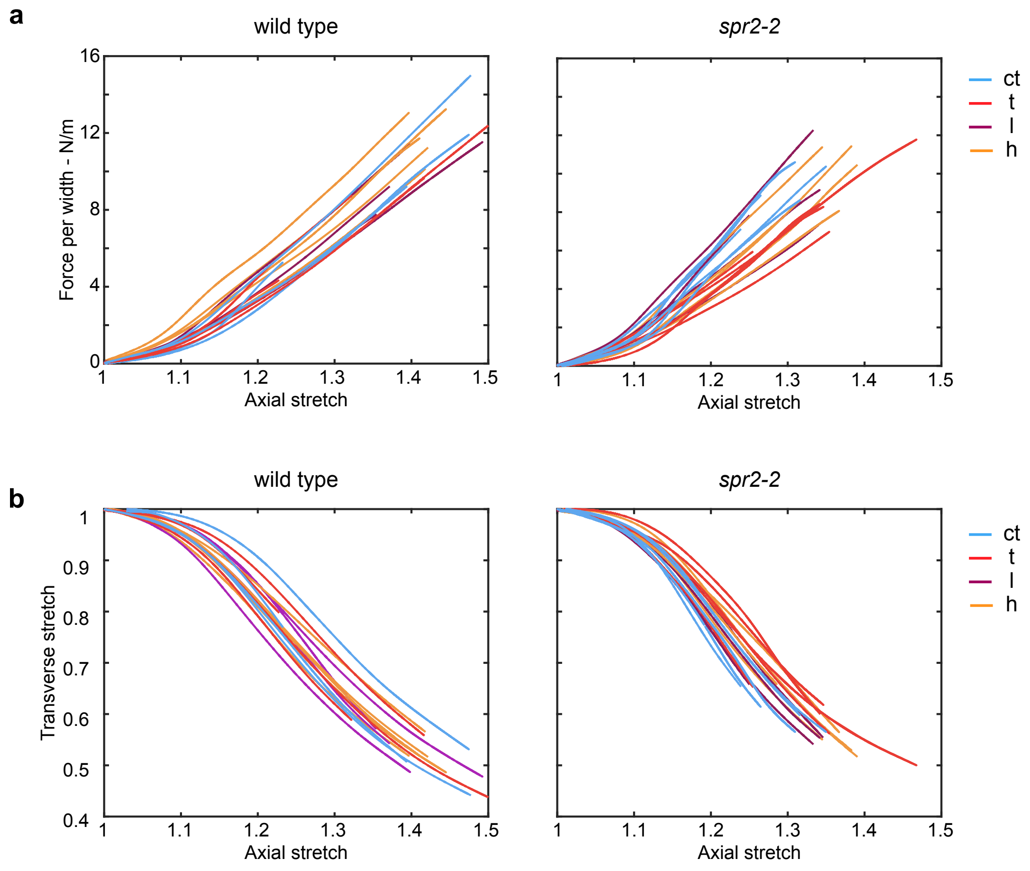

Supplementary Figure 6. **Comparison of monotonic tensile test results for wild type and *spr2-2* of 12d in four orientations:** counterclockwise tilted 45 degrees relative to the midrib (ct), clockwise tilted 45 degrees relative to the midrib (t), proximal-distal (l), and medial-lateral (h): **a** force per width versus axial stretch curves and **b** transverse stretch versus axial stretch curves.

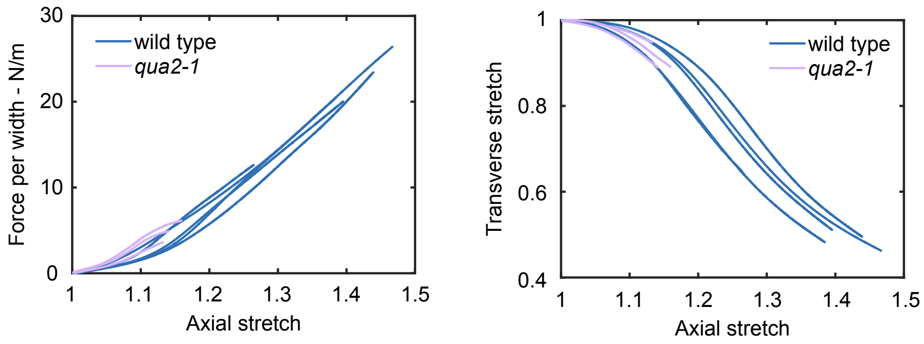

Supplementary Figure 7. **Comparison of monotonic tensile test results for wild type and *qua2-1*.** Force per width – axial stretch and transverse – axial stretch curve for wild type at 25 days post-germination and *qua2-1* at 26 days post-germination. Note that five curves from wild type are reused from Fig. 3b 25d data.

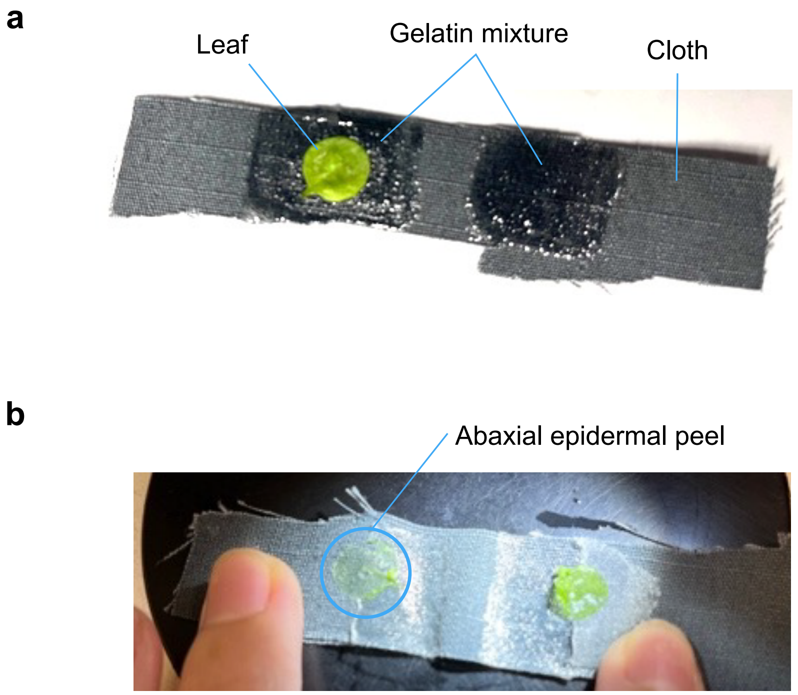

Supplementary Figure 8. **Obtaining leaf epidermal peels using gelatin-coated cloth.** **a** The cloth was coated with gelatin mixture, and the leaf was placed on the gelatin-coated area. **b** After peeling, the abaxial epidermal peel was obtained.

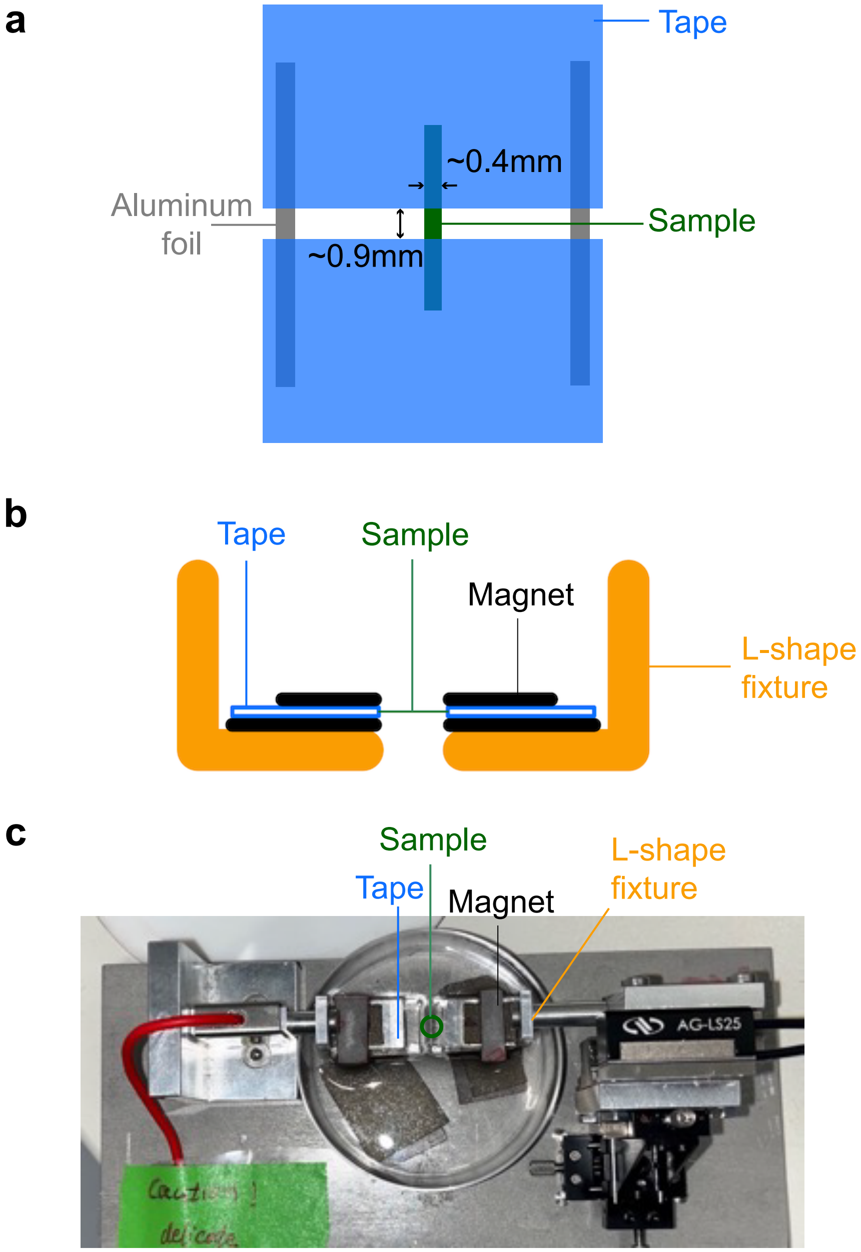

Supplementary Figure 9. **Attaching the sample on the micro-mechanical tensile stage.** **a** Gripping a sample using tapes. **b** Schematic of using magnets to attach the tape-gripped sample to the L-shape fixture of the stage. **c** Top view of the tensile stage with the sample attached.

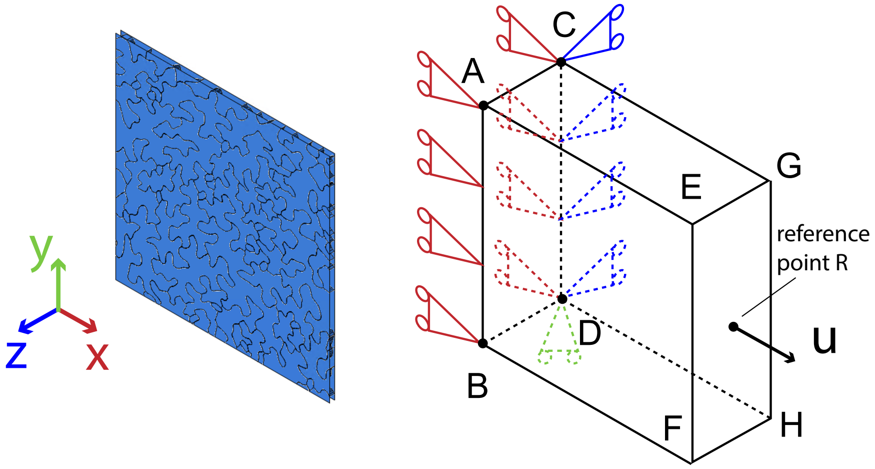

Supplementary Figure 10. **Boundary conditions for FEM simulations.** $x = 0$ on boundary AB, $x = 0$ and $y = 0$ on boundary CD, and point D was fixed. An equation contraint was used to tie boundary EF and GH to the reference point R, and incremental displacement values u were applied to reference point R.

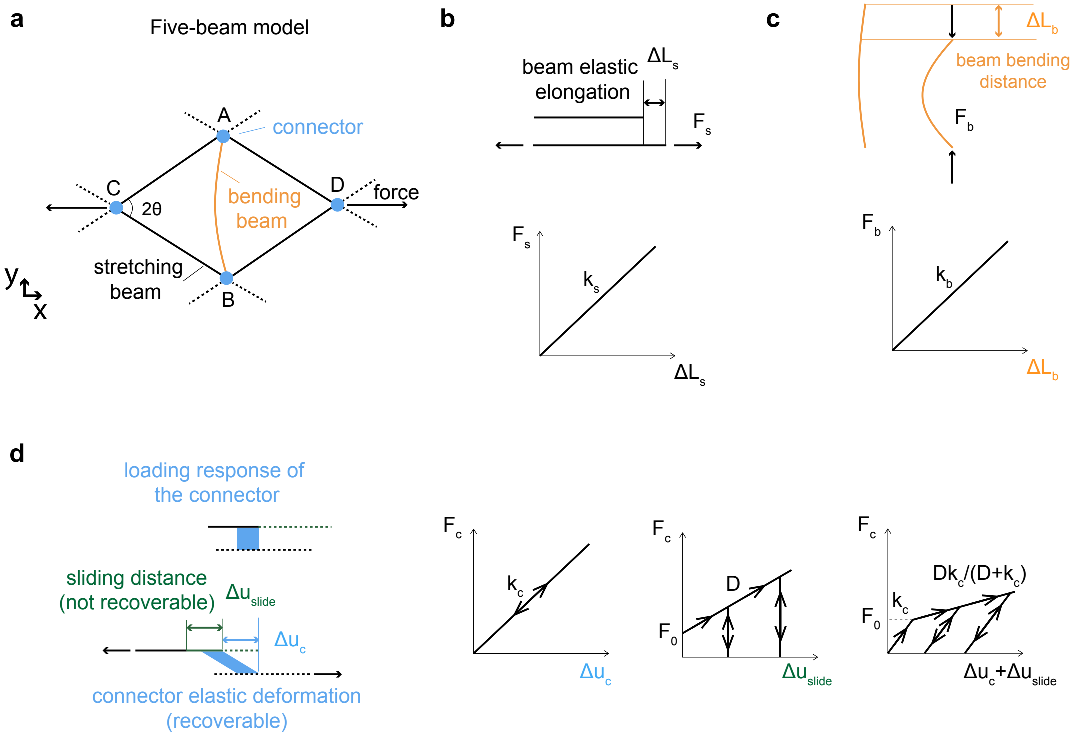

Supplementary Figure 11. **Five-beam model and the force-displacement responses for each components.** **a** schematic of the five model **b** stretching beams **c** bending beam **d** connectors of the five-beam model.

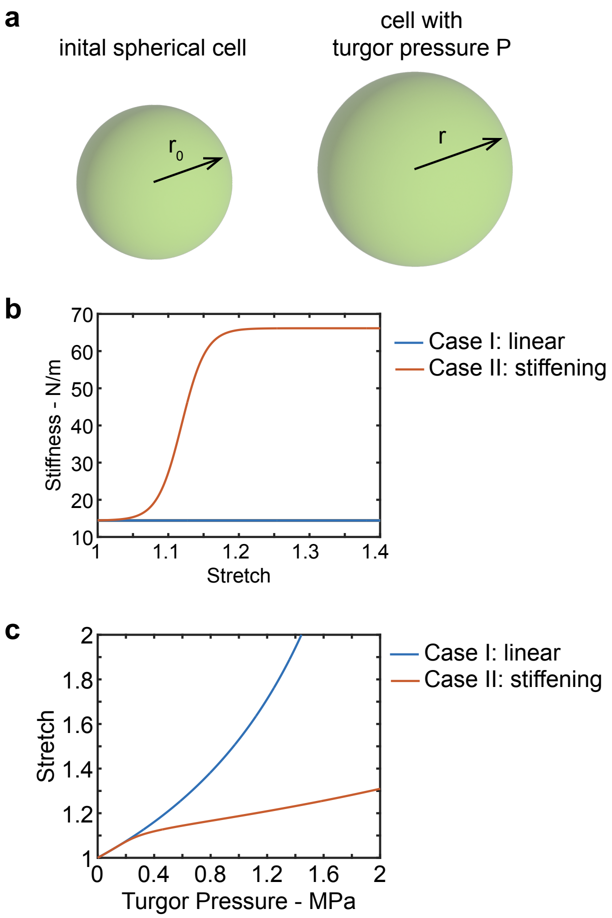

Supplementary Figure 12. **The deformation of spherical cell under turgor pressure for linear behavior and nonlinear stiffening behavior of the cell wall.** **a** initial spherical cell and spherical cell with turgor pressure **b** the stiffness versus the stretch curves **c** stretch of the cell wall versus turgor pressure for linear behavior and nonlinear stiffening behavior.

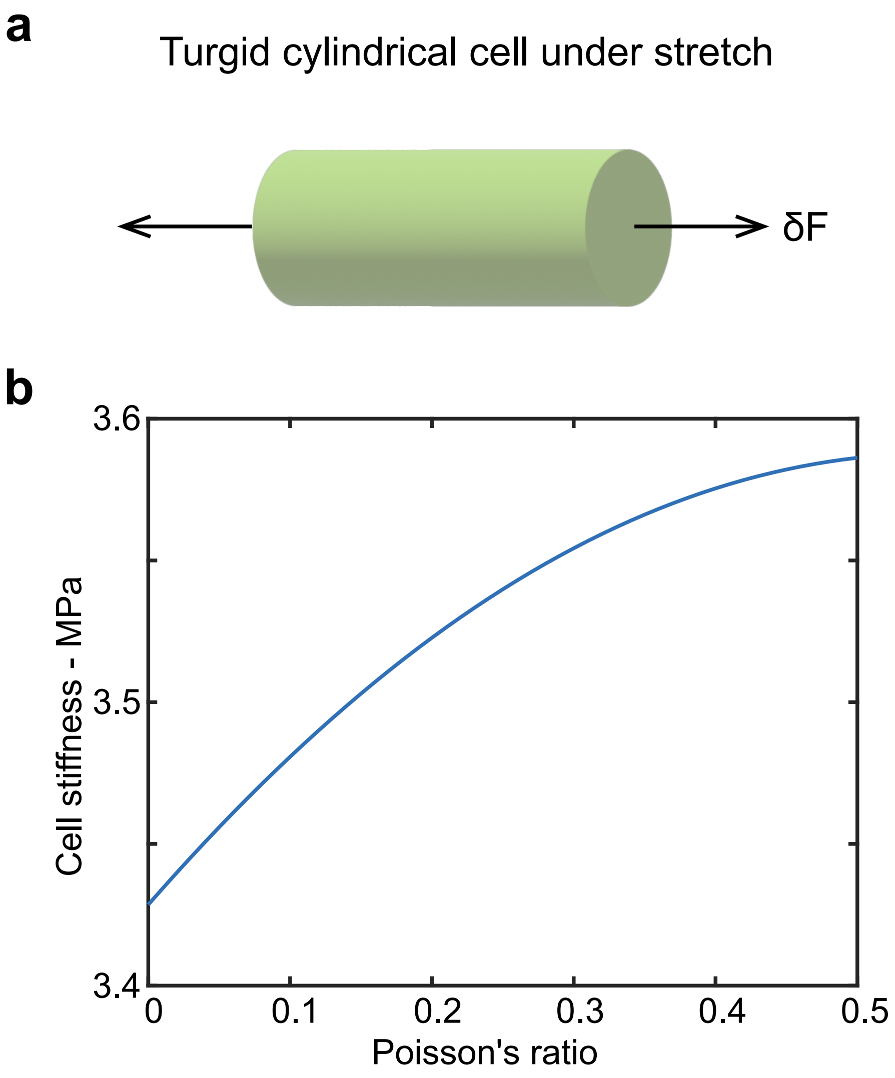

Supplementary Figure 13. **Poisson's ratio influences the stiffness of a turgid cylindrical cell.** **a** A turgid cylindrical cell under axial loading. **b** Stiffness of the cell versus Poisson's ratio curve.

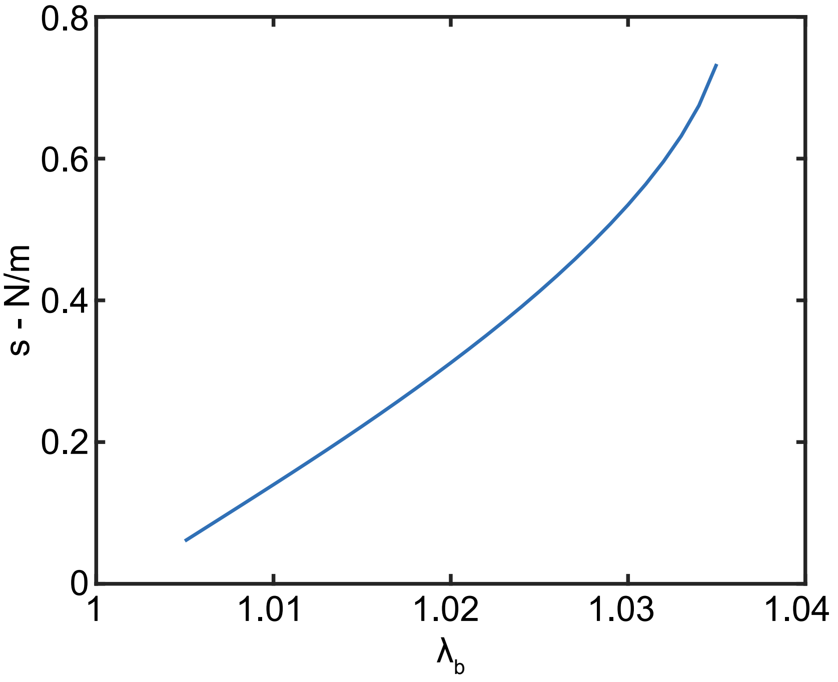

Supplementary Figure 14. **Force per width versus stretch under equal-biaxial loading.**

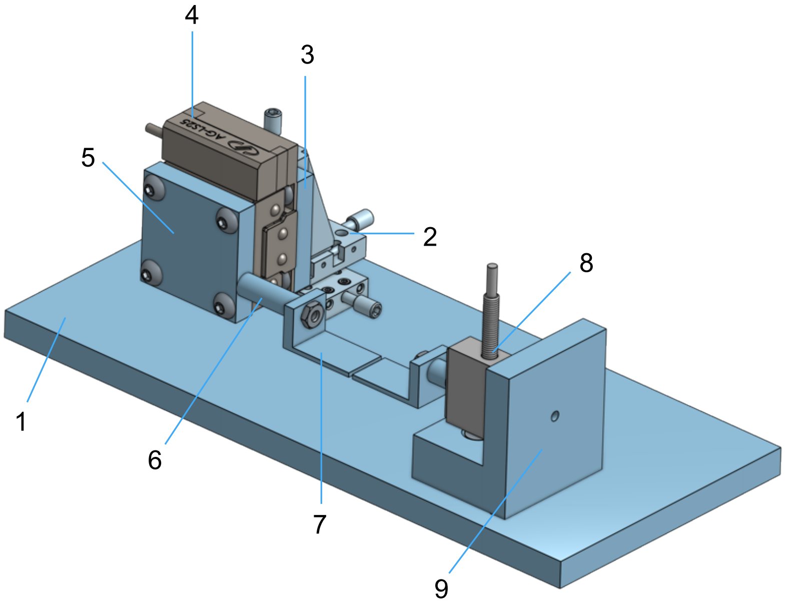

Supplementary Figure 15. **3D CAD drawing of the micro-mechanical tensile stages:** (1) Steel bottom plate; (2) Newport MS-125-XYZ miniature linear stage (Image courtesy of MKS Inc); (3) Aluminum connection plate1; (4) Newport AG-LS25 piezo motor driven linear stage (Image courtesy of MKS Inc); (5) Aluminum connection plate 2; (6) Aluminum rod fixture; (7) L-shape fixture; (8) FUTEK LSB200 load cell (Image courtesy of FUTEK Inc); (9) Aluminum L-shape connection.

Supplementary Table 1. Parameters of material models used in FEM simulations.

Models	C10	C20	C30	D1	D2	D3
neo-Hookean	0.1923	NA	NA	2.4	NA	NA
Yeoh 1	0.1923	-0.002	0.002	2.4	0	0
Yeoh 2	0.1923	0.3846	0	2.4	0	0

Supplementary References

1. Chase P Broedersz and Fred C MacKintosh. Modeling semiflexible polymer networks. *Reviews of Modern Physics*, 86(3):995–1036, 2014.

2. Daniel J Cosgrove. Plant cell growth and cell wall enlargement. *Encyclopedia of Life Sciences (eLS)(Online)*, 2(11), 2022.

3. Anne Mie C Emons and Bela M Mulder. The making of the architecture of the plant cell wall: how cells exploit geometry. *Proceedings of the National Academy of Sciences*, 95(12):7215–7219, 1998.

4. Gerhard A Holzapfel. *Nonlinear solid mechanics: a continuum approach for engineering science*. Kluwer Academic Publishers Dordrecht, 2002.

5. Kaare H. Jensen and Yoèel Forterre, editors. *Soft Matter in Plants: From Biophysics to Biomimetics*. Number 15 in Soft Matter Series. Royal Society of Chemistry, London, 2023.

6. James A. Lockhart. An analysis of irreversible plant cell elongation. *Journal of Theoretical Biology*, 8(2):264–275, March 1965.

7. Grégory Mouille, Marie-Christine Ralet, Céline Cavelier, Cathlene Eland, Delphine Effroy, Kian Hématy, Lesley McCartney, Hoai Nam Truong, Virginie Gaudon, Jean-François Thibault, Alan Marchant, and Herman Höfte. Homogalacturonan synthesis in Arabidopsis thaliana requires a Golgi-localized protein with a putative methyltransferase domain. *The Plant Journal*, 50(4):605–614, 2007.

8. Catalin R Picu. *Network materials: structure and properties*. Cambridge University Press, 2022.

9. P. A. Roelofsen and A. L. Houwink. Architecture and growth of the primary cell wall in some plant hairs and in the phycomyces sporangiophore. *Acta Botanica Neerlandica*, 2(2):218–225, May 1953.

10. Martin H Sadd. *Elasticity: theory, applications, and numerics*. Academic Press, 2009.

11. Larry A Taber. *Nonlinear Theory Of Elasticity: Applications In Biomechanics (Revised Edition)*. World Scientific, 2023.

12. Stéphane Verger, Yuchen Long, Arezki Boudaoud, and Olivier Hamant. A tension-adhesion feedback loop in plant epidermis. *eLife*, 7:e34460, April 2018.

13. Tian Zhang, Yunzhen Zheng, and Daniel J Cosgrove. Spatial organization of cellulose microfibrils and matrix polysaccharides in primary plant cell walls as imaged by multichannel atomic force microscopy. *The Plant Journal*, 85(2):179–192, 2016.
