# Supplementary material for: Fibrous network nature of plant cell walls enables tunable mechanics for development
Source: Nat Commun. 2025 Aug 14;16:7565. doi: 10.1038/s41467-025-62844-1 (PMC12354786; doi:10.1038/s41467-025-62844-1)
Supplement: Supplementary file 2 — Description of Additional Supplementary Information [file 41467_2025_62844_MOESM2_ESM.pdf]

Description of Additional Supplementary Files

File Name: Supplementary Movie 1
Description: **Digital image correlation tracking on the stretching sample.** The sample was coated with green fluorescent beads, and the heat map shows the percentage of deformation in the stretch direction.

File Name: Supplementary Movie 2
Description: **Finite element simulation of stretching single plate.** The heat map shows the stress level in the stretching direction.

File Name: Supplementary Movie 3
Description: **Finite element simulation of stretching cellular structure.** The heat map shows the stress level in the stretching direction.

File Name: Supplementary Software 1
Description: **Custom MATLAB code to implement the affine model and five-beam model.**

File Name: Supplementary Software 2
Description: **Source file for finite element simulation of cellular structure.**
